# A Case Report on Gastric Lipoma: An Unusual Etiology of Gastrointestinal Bleeding

**DOI:** 10.7759/cureus.76699

**Published:** 2024-12-31

**Authors:** Usamah Al-Anbagi, Zaid A Aljuboori, Abdulqadir J Nashwan, Aram Salehi

**Affiliations:** 1 Internal Medicine Department, Hamad Medical Corporation, Doha, QAT; 2 Nursing and Midwifery Research Department, Hamad Medical Corporation, Doha, QAT

**Keywords:** esophagogastroduodenoscopy (ogd), gastric lipoma, gastrointestinal bleeding, melena, robotic gastric surgery

## Abstract

Gastric lipomas are rare, benign tumors of the stomach. They are often asymptomatic and are typically discovered incidentally during imaging studies or endoscopic evaluations. When symptomatic, they may present with nonspecific gastrointestinal (GI) symptoms such as abdominal pain, bleeding, or obstruction. We report a case of a 60-year-old male patient who presented with melena, abdominal pain, and fatigue. Severe anemia was detected, and computed tomography (CT) abdomen and pelvis with contrast revealed a 6.5 cm subepithelial mass in the stomach, initially suspected to be a liposarcoma. However, a biopsy confirmed a benign gastric lipoma. The patient underwent a successful robotic gastric wedge resection and recovered uneventfully. This case highlights the importance of considering gastric lipoma as a rare but significant cause of GI bleeding. Accurate diagnosis through endoscopic evaluation and imaging, followed by curative surgical resection, can lead to favorable outcomes.

## Introduction

Gastric lipomas are rare, benign tumors of mature adipose tissue, representing less than 3% of all benign gastric tumors [1,2]. They are primarily located in the submucosa, particularly in the gastric antrum, and are typically asymptomatic when small [3-5]. However, larger tumors can cause symptoms such as abdominal pain, gastrointestinal (GI) bleeding, or obstruction. Diagnosis of gastric lipomas is challenging due to their submucosal origin and nonspecific symptoms. Still, imaging techniques like computed tomography (CT) and endoscopy are the primary diagnostic tools. CT is particularly useful due to its ability to reveal the fatty composition of the tumors [6], with characteristic findings such as a well-demarcated low-density oval lesion.

Although lipomas can occur throughout the GI tract, the antrum is the most common site of gastric lipoma [7]. The stomach is a less common site, with only 217 cases reported in the literature as of 2021 [7], with very few cases presenting with massive GI bleeding being published [8]. Most gastric lipomas are detected incidentally during imaging for other conditions. However, symptomatic cases often involve tumors larger than 2 cm, which can present with complications such as intussusception or GI hemorrhage. These tumors are more frequently diagnosed in the fifth or sixth decade of life, with no significant sex predilection [9].

The management of gastric lipomas depends on their size and symptoms. While small, asymptomatic lipomas may not require intervention, larger or symptomatic lipomas typically do. Treatment options range from endoscopic excision for smaller lesions to surgery for larger ones. Sometimes, lipomas can mimic other gastric conditions (e.g., gastric adenocarcinoma, gastric lymphoma, and peptic ulcer disease with a mass effect), making accurate diagnosis critical to avoid unnecessary surgical interventions [10-11].

## Case presentation

A 60-year-old male patient presented with a five-day history of melena and a three-day history of abdominal pain, fatigue, and orthostatic dizziness. The patient reported a sudden onset of persistent melena with progressively worsening symptoms. He denied any bleeding from other sites, weight loss, or loss of appetite. He had no history of nonsteroidal anti-inflammatory drug (NSAID) or anticoagulant use, and his past medical history was unremarkable.

On examination, the patient appeared alert but fatigued. He was pale and did not exhibit jaundice. Abdominal examination revealed epigastric tenderness without guarding, rigidity, or rebound tenderness. A digital rectal examination confirmed melena. No hepatosplenomegaly or abdominal distension was noted. Cardiovascular and respiratory examinations were normal, with clear lungs and heart sounds.

Laboratory results revealed significant anemia, with an initial hemoglobin of 2.9 g/dL, which improved to 6.9 g/dL after a four-unit blood transfusion. The RBC count was 1.2 x 10^6/µL, with microcytosis (MCV 81 fL) and elevated reticulocytes (10.9%), suggesting active red cell production. Coagulation studies were normal (international normalized ratio (INR), 1.2; prothrombin time (PT), 13.9 seconds; APTT 27.4 seconds). Urea was elevated at 8.7 mmol/L, indicating active GI bleeding (the urea/creatinine ratio is 22) (Table 1).

**Table 1:** Laboratory investigations HbA1c: hemoglobin A1c; AST: aspartate aminotransferase; ALT: alanine aminotransferase; TSH: thyroid-stimulating hormone; FT3: free triiodothyronine; CA-125: cancer antigen 125; CA 15-3: cancer antigen 15-3; CA 19-9: cancer antigen 19-9; CEA: carcinoembryonic antigen; AFP: alpha-fetoprotein

Parameters	On admission	On discharge	Reference values
Total leukocytes (x10^3/uL)	23	13	(4-10)
Hemoglobin (gm/dL)	2.9	8.8	(13-17)
Mean corpuscular volume (fL)	81.1	82.7	(83-101)
Mean corpuscular hemoglobin (pg)	21.8	25.4	(27-32)
Platelet (x10^3/uL)	327	465	(150-410)
Reticulocytes (%)	10.9	-	(0.5-2.5)
Serum potassium K (mmol/L)	4.1	4	(3.5-5.3)
Serum sodium (mmol/L)	140	140	(133-146)
Serum calcium (mmol/L)	2.28	-	(2.2-2.6)
Serum urea (mmol/L)	8.7	5.2	(2.5-7.8)
Serum creatinine (umol/L)	96	86	(62-106)
HbA1c (%)	5.9	-	<5.7%
Serum albumin (gm/L)	25	26	(35-50)
Serum total protein (gm/L)	52	63	(60-80)
AST (IU/L)	10	24	(0-41)
ALT (IU/L)	6	24	(0-41)
Alkaline phosphatase (U/L)	65	85	(40–129)
TSH (mIU/L)	4.6	-	(0.3-4.2)
FT3 (pmol/L)	13.2	-	(11-23.3)
Serum total bilirubin (mg/dl)	<2	<2	(0-21)
Serum chloride (mmol/L)	107	105	(95-108)
Serum Iron (umol/L)	1	-	(6-35)
Folate (nmol/L)	35	-	(13-104)
Vitamin B12	135	-	(145-569)
CA-125 (IU/mL)	3.7	-	(0-6)
CA 15-3 (U/mL)	11.9	-	(0-35)
CA 19-9 (U/mL)	3.4	-	(0-34.5)
CEA (U/mL)	0.9	-	(0-27)
AFP (ug/L)	<1	-	(0-5)

**Figure 1:**
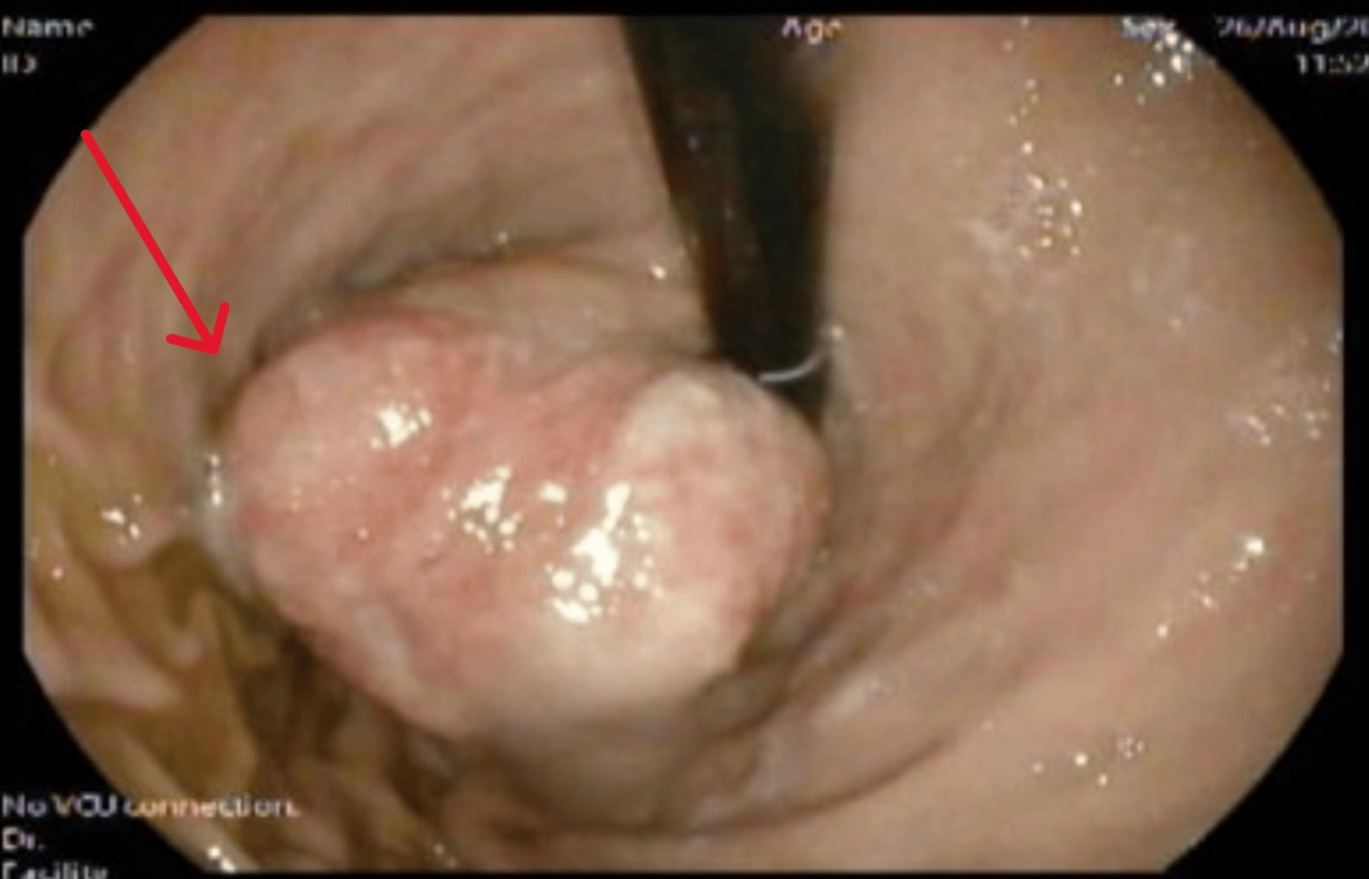
Large subepithelial mass lesion at the cardia Esophagogastroduodenoscopy (EGD) showing a mass in the gastric fundus (red arrow)

An esophagogastroduodenoscopy (EGD) revealed a 7 cm subepithelial mass below the gastroesophageal junction, causing partial obstruction without ulcers or active bleeding; the mass caused a partial obstruction of the gastroesophageal junction, though the passage was still possible. It occupied most of the fundus but originated from the cardia. The overlying mucosa appeared normal with some sloughing, and no ulcers or active bleeding were observed. Biopsies were obtained (Figure 1).

**Figure 2:**
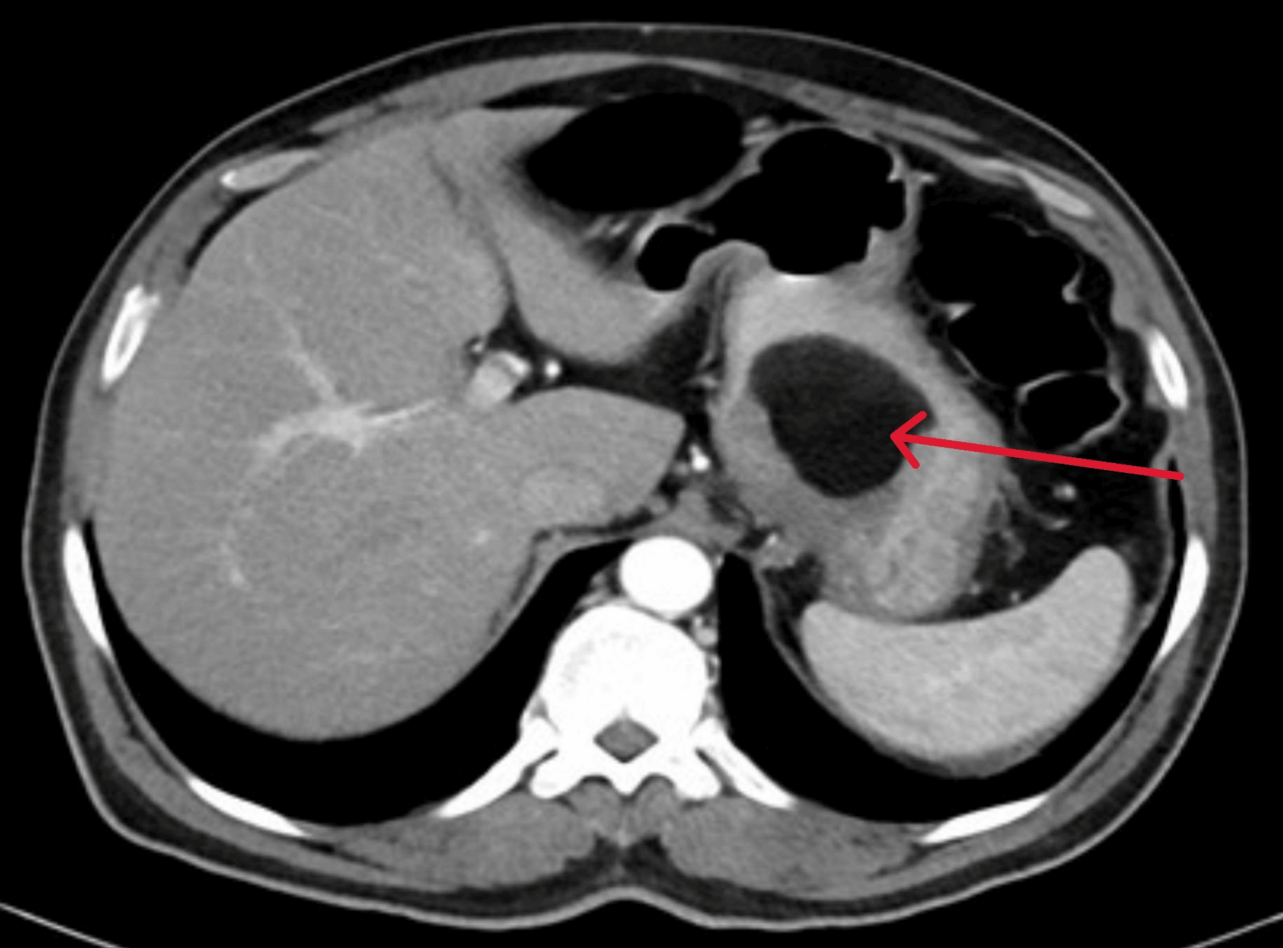
CT scan-axial view, fat-containing intramural subepithelial mass lesion CT scan (axial view) showing a large mass lesion in the fundus and cardia of the stomach, measuring 6.5 x 6.2 x 5.4 cm (red arrow)

**Figure 3:**
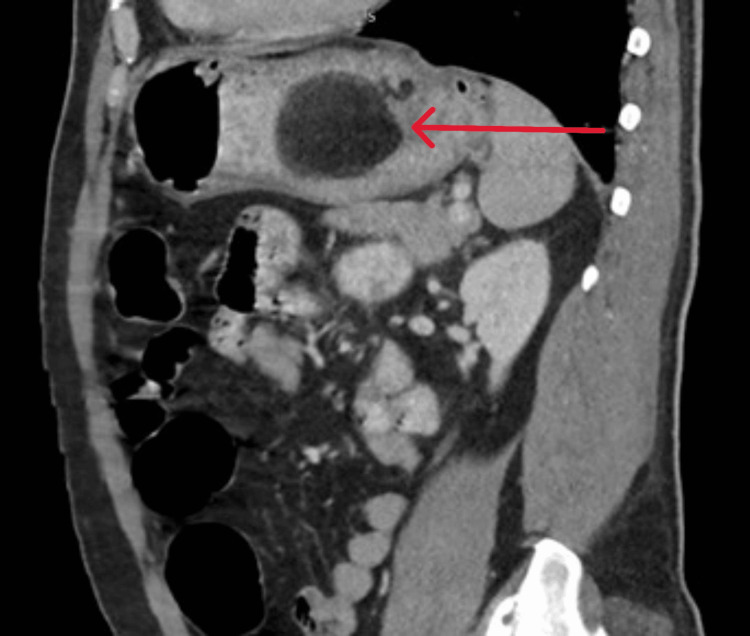
CT scan-sagittal view, fat-containing intramural subepithelial mass lesion CT scan (sagittal view), another view of the gastric mass (red arrow)

A CT abdomen and pelvis with contrast revealed a large submucosal mass lesion noted in the fundus and cardia of the stomach measuring 6.5 x 6.2 x 5.4 cm described as a fat-containing intramural subepithelial mass lesion with septations and soft tissue components. The overlying mucosa was unremarkable, and no metastasis was detected. Features were consistent with a suspected intermediate-grade liposarcoma (Figures 2-3).

Histopathological analysis of the biopsy showed fragments of mature adipose tissue with fat necrosis and ulcer slough, consistent with a gastric lipoma. There was no evidence of malignancy or intestinal metaplasia.

**Figure 4:**
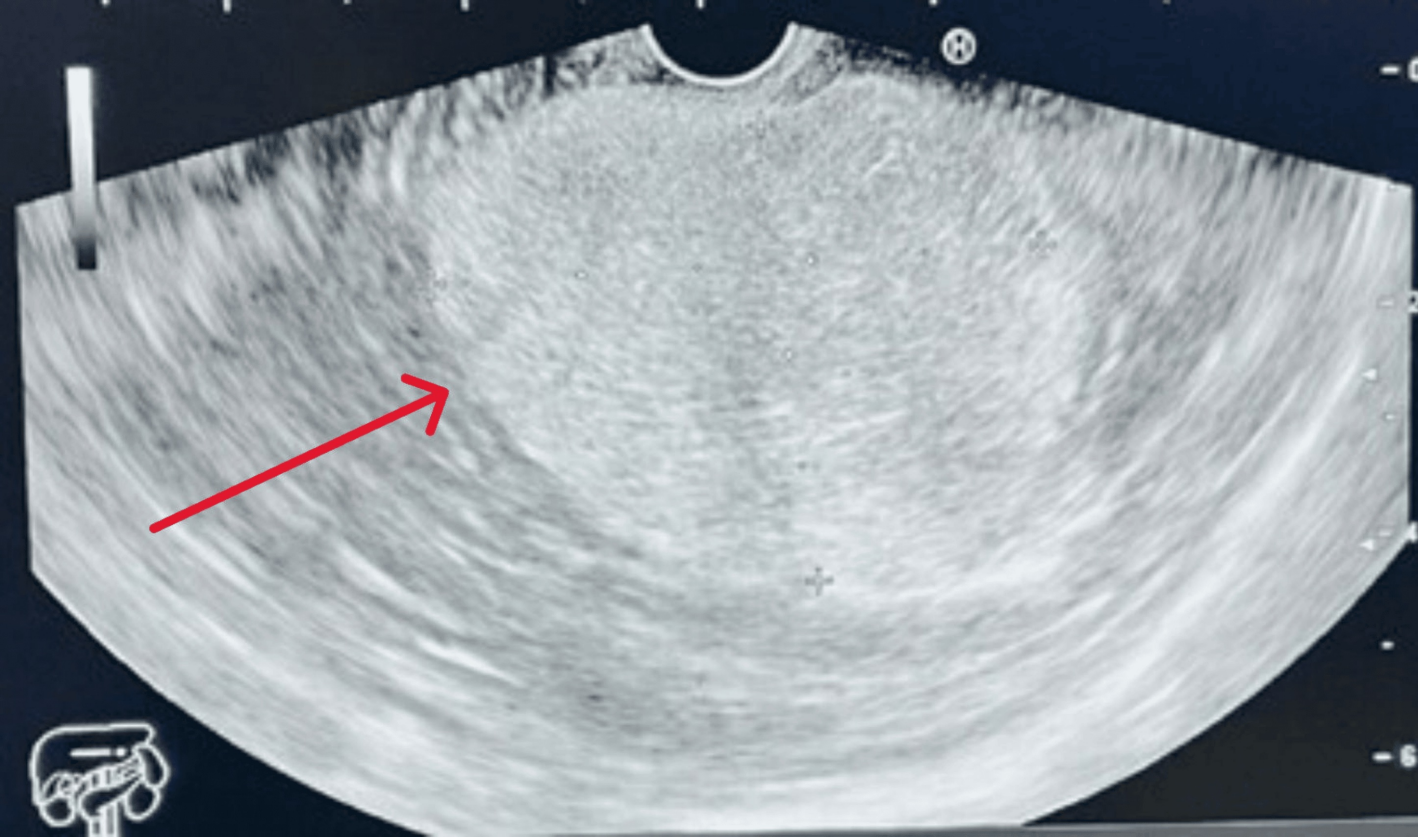
EUS, A 53 X 39 mm well defined, homogenous, hyperechoic subepithelial lesion EUS: endoscopic ultrasound EUS showing a well-defined hyperechoic mass, measuring 53 x 39 mm (red arrow)

The patient was discharged from internal medicine and referred to gastrointestinal surgery and oncology for outpatient follow-up. An endoscopic ultrasound (EUS) performed by an upper GI consultant with robotic surgery expertise confirmed the diagnosis (Figure 4). Subsequently, the case was referred to the multidisciplinary team (MDT).

The MDT recommended curative treatment involved radical surgery. The patient underwent robotic gastric wedge resection of the gastric lipoma. His recovery was uneventful after the surgery, and he was scheduled for a follow-up. The histopathology report of the resected tissue confirmed the diagnosis of a lipoma, which was completely excised with negative margins. He is planned for regular follow-ups at the outpatient clinic.

## Discussion

Gastric lipomas are rare, benign stomach neoplasms, accounting for less than 3% of all benign gastric tumors and 5% of GI lipomas. They consist primarily of mature adipose tissue surrounded by a fibrous capsule and are usually asymptomatic, with their diagnosis often incidental [12]. Larger lipomas, especially those over 2 cm, may cause symptoms such as GI bleeding, obstruction, and abdominal pain due to ulceration of the overlying mucosa. Though the malignant transformation of gastric lipomas has not been reported, larger lesions can lead to serious complications, including gastric outlet obstruction or gastroduodenal intussusception, necessitating intervention [13].

In our patient, a gastric lipoma was discovered incidentally during diagnostic evaluation for melena. This case adds to the body of literature reporting symptomatic presentations despite the typically benign nature of these tumors. Unlike most lipomas, which are asymptomatic, the large size of this lipoma likely contributed to the patient's symptoms. Bleeding is a common presentation in cases where lipomas ulcerate. Still, the peculiarity of this case lies in the rare presentation of gastric lipoma without ulceration, leading to severe anemia and upper GI bleeding [3,14]. We carefully excluded all other possible local GI causes, concluding that the gastric lipoma was the primary source of the bleeding. The possible causes of bleeding, in this case, could be mechanical disruption, leading to local irritation or pressure on the surrounding gastric mucosa, the vascularity of the lipoma, which may cause bleeding even without ulceration, or trauma/friction if the lipoma is located in an area subject to mechanical stress, such as during peristalsis or food bolus passage.

Endoscopic and radiologic evaluations are crucial in diagnosing gastric lipomas. Imaging modalities, mainly CT scans, provide a more definitive diagnosis. In this case, CT imaging revealed a fat-containing intramural subepithelial mass lesion with septations and soft tissue components, confirming the diagnosis of a lipoma. EUS further supported the diagnosis by demonstrating a well-defined, homogeneous, hyperechoic subepithelial lesion [15].

Management of gastric lipomas depends on their size, location, and symptomatology. Smaller, asymptomatic lipomas can be managed conservatively with periodic follow-up [16]. However, larger or symptomatic lipomas, like in our case, often require intervention. Surgical resection is generally recommended for lipomas causing significant symptoms or complications [17-18]. While endoscopic removal may be possible for smaller lesions, larger tumors, or those located near the pylorus, may require more invasive techniques. In our case, the patient underwent successful robotic gastric wedge resection, which treated the lipoma and prevented future complications. This minimally invasive approach provided curative treatment with a favorable recovery and cosmetic outcome.

The case of our patient emphasizes the importance of recognizing the potential for symptomatic presentations in gastric lipomas, particularly those that exceed 2 cm in size. Although rare, these benign tumors can lead to severe GI complications. Early diagnosis through endoscopic and imaging techniques and appropriate surgical intervention can prevent further morbidity. Our patient's favorable outcome after robotic surgery highlights the efficacy of minimally invasive techniques in managing large gastric lipomas. The case also highlights that despite the worrying symptoms and the findings on the EGD, when it comes to informing the patients about the working diagnosis, we could still be hopeful that the nature of the disease is benign and full recovery is possible.

## Conclusions

This case emphasizes the significance of thorough diagnostic evaluation in patients presenting with GI bleeding, particularly when uncommon causes such as gastric lipoma are involved. Early identification using endoscopic and imaging techniques, followed by multidisciplinary management and appropriate surgical intervention, can lead to successful curative outcomes. The patient's case demonstrates the effectiveness of robotic gastric wedge resection for complete removal with negative margins, ensuring an uneventful recovery and excellent prognosis.

## References

[1] Sabbah M, Nakhli A, Helal I, Bellil N, Ouakaa A, Gargouri D (2020). Gastrointestinal bleeding as an initial manifestation of gastric lipoma: case report and review of the literature. Clin Case Rep.

[2] Pack GT (1964). Unusual tumors of the stomach. Ann N Y Acad Sci.

[3] Ramdass MJ, Mathur S, Seetahal-Maraj P, Barrow S (2013). Gastric lipoma presenting with massive upper gastrointestinal bleeding. Case Rep Emerg Med.

[4] Suarez Moreno RM, Hernandez Ramirez DA, Madrazo Navarro M, Salazar Lozano CR, Martinez Gen R (2010). Multiple intestinal lipomatosis. Case report. Cir Cir.

[5] Thompson WM, Kende AI, Levy AD (2003). Imaging characteristics of gastric lipomas in 16 adult and pediatric patients. AJR Am J Roentgenol.

[6] Hamdane MM, Brahim EB, Salah MB, Haouas N, Bouhafa A, Chedly-Debbiche A (2013). Giant gastric lipoma mimicking well-differentiated liposarcoma. Pan Afri Med Jour.

[7] Jarrett SA, Tito S, Chan M, Jarrett DE, Lo KB, DePalma R (2024). Gastric lipomas: a case series and review of the literature. Case Rep Gastroenterol.

[8] Kumar S, Kumar A, Dayal M, Prakash V (2021). Gastric lipoma: a rare cause of massive haematemesis. Ann R Coll Surg Engl.

[9] Termos S, Reslan O, Alqabandi O (2017). Giant gastric lipoma presenting as GI bleed: enucleation or resection?. Int J Surg Case Rep.

[10] Saltzman JR, Carr-Locke DL, Fink SA (2005). Lipoma case report. MedGenMed.

[11] Tomofuji K, Watanabe J, Ishida N, Kajiwara S (2017). Gastric liposarcoma resected by laparoscopic total gastrectomy to achieve a wide surgical margin. BMJ Case Rep.

[12] Cherian S, Mishra A, Patel RD, Chikale NP (2012). Gastric lipoma presenting as obstruction: role of intraoperative frozen section in diagnosis. Clin Cancer Investig J.

[13] Cappell MS, Stevens CE, Amin M (2017). Systematic review of giant gastric lipomas reported since 1980 and report of two new cases in a review of 117110 esophagogastroduodenoscopies. World J Gastroenterol.

[14] Krishnaraj B, Dhanapal B, Shankar G, Sistla SC, Galidevara I, Suresh A (2018). Gastric lipoma: a rare cause of haematemesis. Ann R Coll Surg Engl.

[15] Yamamoto T, Imakiire K, Hashiguchi S (2004). A rare case of gastric lipoma with early gastric cancer. Intern Med.

[16] Nasa M, Choksey A, Phadke A, Sawant P (2016). Gastric lipoma: an unusual cause of dyspeptic symptoms. BMJ Case Rep.

[17] Fukuda S, Yamagata R, Mikami T (2003). Gastric lipoma successfully treated by endoscopic unroofing. Dig Endosc.

[18] Ponsaing LG, Hansen MB (2007). Therapeutic procedures for submucosal tumors in the gastrointestinal tract. World J Gastroenterol.

